# Metaviromes of Extracellular Soil Viruses along a Namib Desert Aridity Gradient

**DOI:** 10.1128/genomeA.01470-16

**Published:** 2017-01-12

**Authors:** Olivier Zablocki, Evelien M. Adriaenssens, Aline Frossard, Mary Seely, Jean-Baptiste Ramond, Don Cowan

**Affiliations:** a Centre for Microbial Ecology and Genomics, University of Pretoria, Pretoria, South Africa; b Department of Microbiology and Plant Pathology, University of Pretoria, Pretoria, South Africa; c Department of Genetics, University of Pretoria, Pretoria, South Africa; d Gobabeb Research and Training Centre, Walvis Bay, Namibia; e School of Animal, Plant and Environmental Sciences, University of the Witwatersrand, Johannesburg, South Africa; f Institute for Microbial Biotechnology and Metagenomics, University of the Western Cape, Bellville, South Africa

## Abstract

The Namib Desert in southwest Africa is hyperarid and composed of distinct microbial communities affected by a longitudinal aridity gradient. Here, we report four soil metaviromes from the Namib Desert, assessed using deep sequencing of metavirome libraries prepared from DNA extracted from gravel plain surface soils.

## GENOME ANNOUNCEMENT

Desert environments are one of the largest terrestrial ecosystems on Earth, and account for ~33% of terrestrial land masses (1). However, the diversity and ecological functions of viruses in these arid ecosystems are mostly unknown (2). Virus research in arid soils has primarily focused on the taxonomic composition associated with surface soils and soil-lithic interfaces (3–10) and has revealed a wide diversity of virus taxa, mostly composed of tailed phage families (order: *Caudovirales*).

The Namib Desert is considered hyperarid, and soils are subjected to the effects of a longitudinal aridity (and water availability) gradient, derived from sporadic fog and rainfall events (11). An inverse fog-rainfall gradient has been shown to significantly contribute to the spatial composition and community structure of open surface soil and rock-associated (hypolith) microbial communities (12). However, the extent of extracellular viral diversity remains unknown.

Surface soil samples were collected during April 2013 at 30 km intervals along the C14 highway, extending across the Namib Desert from Walvis Bay toward Windhoek. Five hundred grams of surface soil (0 to 2 cm depth) was collected at each site (GPS coordinates: “NAM10”: 22 58.773 S, 014 34.994 E; “NAM40”: 23 01.080 S, 014 51.587 E; “NAM70”: 23 06.004 S, 015 07.676 E; “NAM100”: 23 14.776 S, 015 16.881 E; “NAM130”: 23 19.243 S, 015 37.370 E) and processed for DNA extraction as previously described (13). Library construction for sequencing was done using the Ion Xpress Plus and Ion Plus library preparation for the AB library builder system (publication number MAN0006946). Template amplification was performed using the Ion OneTouch 2 System (OT2) Ion PI Hi-Q OT2 200 kit (number MAN0010857). The metavirome libraries were multiplexed and sequenced using the Ion PI Hi-Q sequencing 200 kit (number MAN0010947) using the Ion PI chip kit v3. Sequencing was performed on the Ion Proton platform, located at the Central Analytical Facilities, Stellenbosch University, South Africa.

CLC Genomics version 6.0.1 (CLC, Denmark) was used to curate, bin, and assemble reads using the default parameters. Contigs were uploaded to the MetaVir version 2 server (http://metavir-meb.univ-bpclermont.fr/) for taxonomic assignment and gene annotation (publicly accessible through project “Namib Desert transect metaviromes”). Sequencing of the four metaviromes produced 93,519,306 reads, which resulted in each metavirome data set totaling ~ 22 million reads, with a mean read length of 142.5 bp and a G+C content ranging from 54 to 62%. Across all soil samples, the ratio of sequences with taxonomic assignment (i.e., having a database homologue) ranged from 9.18 to 18.91%, indicative of a highly uncharacterized pool of viral diversity. *Mycobacterium* phages represented the top sequence hits across all soil samples (11.4 to 19.3%), except for NAM40, where *Escherichia* phages were most numerous (18.6%). The next most represented phage isolates across the transect samples were to *Rhodococcus* phages in NAM130 (9.83%), *Bacillus* phages in NAM100 (8%), and *Geobacillus* phages in NAM70 (5.3%). This data provides valuable insights into the virus ecology of desert soils, and highlights the potential for novel virus discovery in a hyperarid desert environment.

### Accession number(s).

The metagenomic reads from the four surface soil samples have been deposited at DDBJ/EMBL/GenBank under the accession no. ERX1230691 to ERX1230694.
